# Population pharmacokinetics of afatinib and exposure-safety relationships in Japanese patients with EGFR mutation-positive non-small cell lung cancer

**DOI:** 10.1038/s41598-019-54804-9

**Published:** 2019-12-03

**Authors:** Keiko Nakao, Shinji Kobuchi, Shuhei Marutani, Ayano Iwazaki, Akihiro Tamiya, Shunichi Isa, Kyoichi Okishio, Masaki Kanazu, Motohiro Tamiya, Tomonori Hirashima, Kimie Imai, Toshiyuki Sakaeda, Shinji Atagi

**Affiliations:** 1 Department of Internal Medicine, National Hospital Organization Kinki-Chuo Chest Medical Center, Osaka, Japan; 2 Department of Pharmacokinetics, Kyoto Pharmaceutical University, Kyoto, Japan; 3 Faculty of Pharmaceutical Sciences, Setsunan University, Osaka, Japan; 4 Department of Thoracic Oncology, National Hospital Organization Kinki-Chuo Chest Medical Center, Osaka, Japan; 5 Department of Thoracic Oncology, National Hospital Organization Osaka Toneyama Medical Center, Osaka, Japan; 6 Department of Thoracic Oncology, Osaka International Cancer Institute, Osaka, Japan; 7 Department of Thoracic Malignancy, Osaka Habikino Medical Center, Osaka, Japan

**Keywords:** Drug development, Targeted therapies

## Abstract

To investigate the exposure–safety relationships of afatinib in Japanese population, we performed population pharmacokinetics (PK) analysis of afatinib in Japanese advanced non-small cell lung cancer patients harboring epidermal growth factor receptor mutation. Plasma samples were collected at 0.5–1, 2–3, 8–12, and 24 h after oral afatinib (40 mg) administration on day 1 and day 8. Plasma afatinib concentrations were determined using high-performance liquid chromatography. Data was analyzed following the population approach and using the software Phoenix^®^ NLME^TM^ Version 7.0 software (Certara USA, Inc., Princeton, NJ, USA). From 34 patients, a total of 354 afatinib plasma concentration values were available for the population PK analysis. Significant covariates in the population PK model included aspartate aminotransferase and creatinine clearance on CL/F, and age and body mass index on V/F. Results of simulation based on final PK model indicated that hepatic impairment had a significant effect on afatinib levels in plasma after multiple dosing. Afatinib trough plasma concentrations on day 8 were higher in patients with adverse events of grade 3 or higher. The population PK analysis showed that hepatic impairment affected afatinib PK parameters and contributed to the high inter-patient variability and high plasma concentrations of afatinib following multiple treatments.

## Introduction

Epidermal growth factor receptor (EGFR) tyrosine kinase inhibitors (TKIs) such as gefitinib, erlotinib, afatinib, and osimertinib are the standard first-line therapies for patients with *EGFR* mutation-positive non-small-cell lung cancer (NSCLC)^1^. Afatinib is a second generation, irreversible ErbB family blocker, which shows inhibitory activity against EGFR, human EGFR 2 (HER2) and 4 (HER4), with IC50 values of 0.5, 14, and 1 nM, respectively^2–4^. Afatinib has also been reported to perform significantly better than standard chemotherapy in improving the response rate (RR) and progression-free survival (PFS) of patients with EGFR mutation-positive advanced NSCLC in a first-line setting^5,6^. A LUX-lung 7 study showed that the PFS, time-to-treatment failure, and RR with afatinib were superior to those with gefitinib in patients with treatment-naive lung adenocarcinoma having EGFR mutation. However, owing to its phase-IIb nature, this study did not have the power to draw any definite conclusions, and there was no significant difference between the two drugs in terms of the overall survival (OS)^7,8^. Recently, the superiority of afatinib to erlotinib was demonstrated in patients with squamous cell lung cancer who had progressed on platinum-based chemotherapy^9^. The most common adverse events (AEs) for afatinib include rash or acne, diarrhea, and stomatitis whereas hepatic impairment occurs less frequently^5–9^. Although these AEs are typically manageable, dose reduction and treatment discontinuation are frequently required. However, comprehensive therapeutic effects are difficult if the drug is not used continuously at its full dose.

A pharmacokinetics (PK) analysis showed that plasma concentrations of afatinib peaked at 3–4 h after administration and declined with a half-life of 37 h at steady state^4^. Afatinib plasma level shows high inter-patient variability^5^. Therefore, monitoring blood afatinib concentration is important for its dose adjustment and treatment continuation.

The population PK approach is often used for PK analysis in a clinical study, as a substitute for or in addition to the standard PK approaches^10^. Freiwald *et al*. studied the population PK of afatinib based on the data from 927 cancer patients including 764 NSCLC patients in 7 Phase II or III studies. Age, smoking history, alcohol consumption, and presence of liver metastases did not show significant impact on the afatinib exposure^11^. However, best of our knowledge, we have only identified one report describing population PK model of afatinib which lacks information on the exposure-safety relationship. The present population PK analysis aimed to investigate the exposure-safety relationships of afatinib in Japanese NSCLC patients with EGFR mutation.

## Results

A total of 354 afatinib plasma concentration values from 34 patients were used for the population PK analysis. The demographic and baseline characteristics of the participants are described in Table 1. Among the patients included in the dataset, two were older than 80 years and two had a body mass index (BMI) of less than 18.5 kg/m^2^. A relatively large inter-individual variability was observed in aspartate aminotransferase (AST) (13–65 IU/L), alanine aminotransferase (ALT) (5–77 IU/L), and creatinine clearance (Ccr) (42.3–131.8 mL/min) levels. Among the patients, four had a AST/ALT of higher than limit normal and ten had a Ccr of less than limit normal (12.4% and 27.7% of all evaluable PK samples from patients with hepatic and renal impairment, respectively).

**Table 1:** Demographic and clinicopathologic characteristics of patients.

Characteristics		n
Sex		34
Male		11
Female		23
Tumor histology		
Adenocarcinoma		34
ECOG score		
0–1		_28_
2		5
3		1
EGFR mutation status		
Ex19 del.		18
Ex19 del.+ T790M		1
Ex19 del. + Ex19 A755G		1
L858R		9
L858R+ Ex18 G719C		1
Ex18 G719S		1
Ex18 G719C		1
Ex20 ins		1
S768I		1
Prior CTX		24
Prior EGFR TKI		21
Value (units)	Mean ± SD	Range
Age (years)	66.8 ± 1.5	45–86
Height (cm)	156.5 ± 1.7	138.0–186.0
Weight (kg)	53.8 ± 1.6	35.5–79.1
BMI (kg/m^2^)	21.9 ± 0.5	15.2–28.1
AST (IU/L)	25.6 ± 2.0	13–65
ALT (IU/L)	19.7 ± 2.6	5–77
Cre (mg/dL)	0.64 ± 0.03	0.39–1.13
Ccr (mL/min)	80.8 ± 3.8	42.3–131.8

Each value represents mean ± SD with the range in parentheses, unless specified otherwise. Ccr value was calculated using the Cockroft-Gault method.

*ALT*, alanine aminotransferase; *AST*, aspartate aminotransferase; *BMI*, body mass index; *Ccr*, creatinine clearance; *Cre*, serum creatinine; *CTX*, chemotherapy; *ECOG*, Eastern Cooperative Oncology Group; *EGFR*, epidermal growth factor; *TKI*, tyrosine kinase inhibitor; *SD*, standard deviation.

Matrix diagram was performed by the IBM SPSS Statistics 23 software (SPSS Inc., Chicago, IL, USA) and the results have been described in Fig. S1 (see Supplementary Fig. S1). There were several significant relationships between the covariates, indicating that related covariates may have interaction when adding them into the population PK model.

### Population pharmacokinetics model

Fig. S2 shows the plasma concentration-time profiles of afatinib in patients with EGFR mutation-positive NSCLC (see Supplementary Fig. S2). Table S1 shows the PK parameters obtained by the non-compartment analysis (see Supplementary Table S1). A high inter-patient variability in PK parameters was observed on Day 1 and Day 8. A one-compartment model with first-order absorption and elimination adequately described the PK profile of afatinib. Table 2 shows the final population PK parameter estimates along with their precision, and the results of the bootstrap validation procedure. For a typical patient (i.e., age 66.7 years; BMI 21.8 kg/m^2^; AST 25.3 IU/L; Ccr 79.9 mL/min) who received 40 mg afatinib once daily, the typical CL/F was 20.0 L/h and V/F was 795.8 L. The CV% for each population mean parameter (fixed effects) estimate was ≤43.5%. Inter-individual variability (random effects) was estimated for all parameters (i.e., *k*a, CL/F, and V/F) in the one-compartment model. Relatively large inter-individual variability was observed in *k*a (*ω*_*k*a_ = 92.9%) and CL/F (*ω*_CL/F_ = 76.6%). The CV% for each inter-individual variability parameter and the residual variability (intra-individual variability) was ≤30.3% and 7.2%, respectively. Each median value of the population PK parameter estimates obtained using the bootstrap procedure was similar to that obtained from the original data set, indicating that the final model adequately estimated the model parameters. The 97.5^th^ confidence intervals obtained from bootstrap analysis include the zero. This result was expected because the number of patients (n = 34) was small and large sample size is needed to more precisely estimate inter-individual variability parameters.

**Table 2:** Population pharmacokinetics parameters of afatinib, and results of the bootstrap validation procedure.

Parameters	Final model		Bootstrap (n = 1000)	
	Estimate	CV%	Median	2.5^th–^97.5^th^ percentiles
**Population mean parameters**				
*k*_a_ (_1/h)_	0.60	18.5	0.60	0.40–0.88
CL/F = *θ*_CL_ * (1 + (Ccr–79.9)*θ_Ccr_) * (1 + (AST–25.3) * *θ*_AST_)				
*θ*_CL_ (L/h)	20.0	13.4	19.9	15.3–26.3
*θ*_Ccr_ (min/mL)	0.0013	22.2	0.0013	−0.0047–0.0119
*θ*_AST_ (L/IU)	−0.016	27.5	−0.016	−0.022–0.004
V/F = *θ*_V_ * (1 + (BMI–21.8)*θ_BMI_) * (1 + (Age–66.7)*θ_Age_)				
*θ*_V_ (L)	795.8	9.5	789.0	626.0–971.3
*θ*_BMI_ (m^2^/kg)	0.019	42.8	0.019	−0.031–0.078
*θ*_Age_	−0.004	43.5	−0.004	−0.020–0.018
**Inter-individual variability**				
*ω*_*k*a_ (%)	92.9	30.3	91.8	61.7–138.2
*ω*_CL/F_ (%)	76.6	18.2	74.1	53.4–91.3
*ω*_V/F_ (%)	52.8	12.2	52.3	36.9–66.1
**Residual variability**				
_*σ* (%)_	31.7	7.2	31.4	27.2–35.7

*ALT*, alanine aminotransferase; *AST*, aspartate aminotransferase; *BMI*, body mass index; *Ccr*, creatinine clearance; *CL*, clearance; *Cre*, serum creatinine; *ka*, absorption rate constant; *V*, distribution volume.

The significant covariates in the population PK model included AST and Ccr on CL/F, as well as age and BMI on V/F. This result of covariate analysis indicated that hepatic and renal impairment had a significant effect on the PK parameters of afatinib. No other evaluated covariate was found to significantly affect the PK parameters of afatinib.

**Figure 1:**
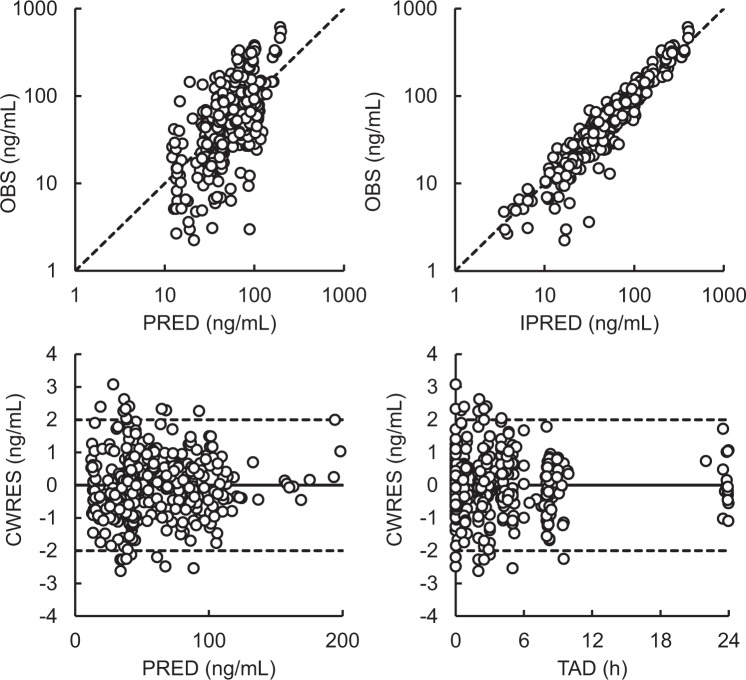
Final pharmacokinetics model diagnostic plots of the observed versus predicted concentrations and the conditional residuals versus predicted concentrations. CWRES, conditional weighted residuals; IPRED, individual predicted concentration; OBS, observed concentration; PRED, population predicted concentration; TAD, time after dose

**Figure 2:**
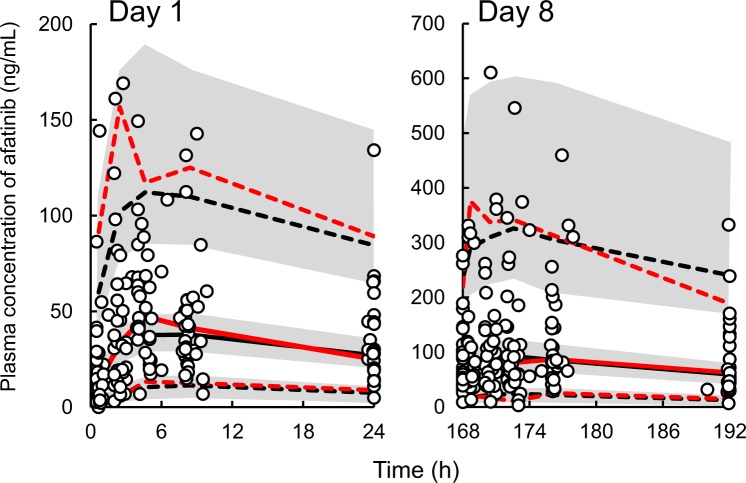
Visual predictive check plot for the final population model of afatinib. The solid line represents the median, whereas the dashed lines represent the 95^th^ percentile (upper) and 5^th^ percentile (lower) of the observed data (red) and 1000 data sets simulated using the final population model (blue). The shaded areas represent the 95% confidence intervals of their respective predictions. The dots represent the observed data.

Figure 1 shows the goodness-of-fit plots, and Fig. 2 presents the results of the visual predictive check of the final population PK model. The predicted concentrations matched the observed data satisfactorily. The CWRES distribution closely resembled a normal distribution and was symmetrically distributed on zero across PRED values and times after dose. The visual predictive check showed that most of the observed data were within 95% prediction percentiles. Therefore, these results suggest that the population PK model fitted the observed data, adequately describing the population and individual plasma concentrations of afatinib.

### Model-based simulation

**Figure 3:**
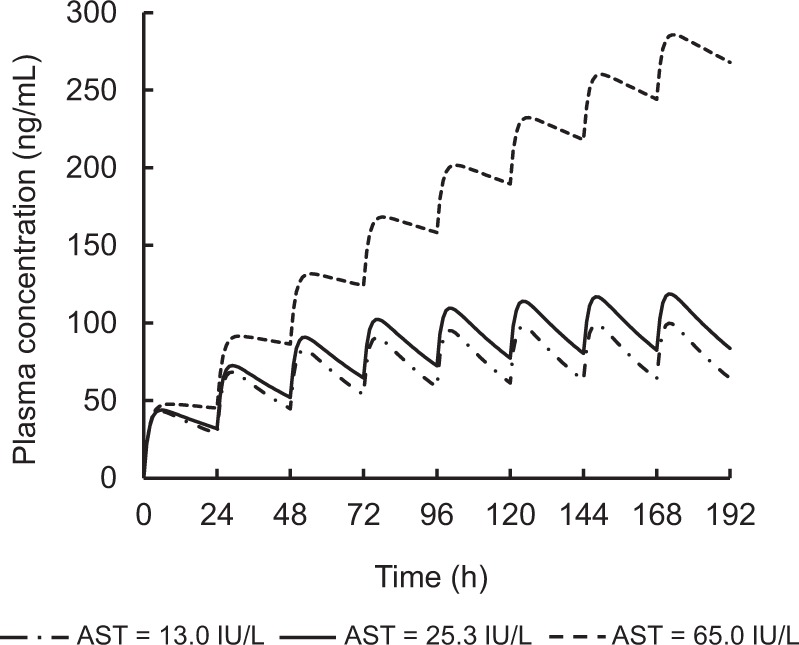
Simulated population mean plasma concentration profiles of afatinib after oral administration at 40 mg/day for different AST values in a typical reference patient (age 66.7 years; BMI 21.8 kg/m^2^; Ccr 79.9 mL/min).

**Figure 4:**
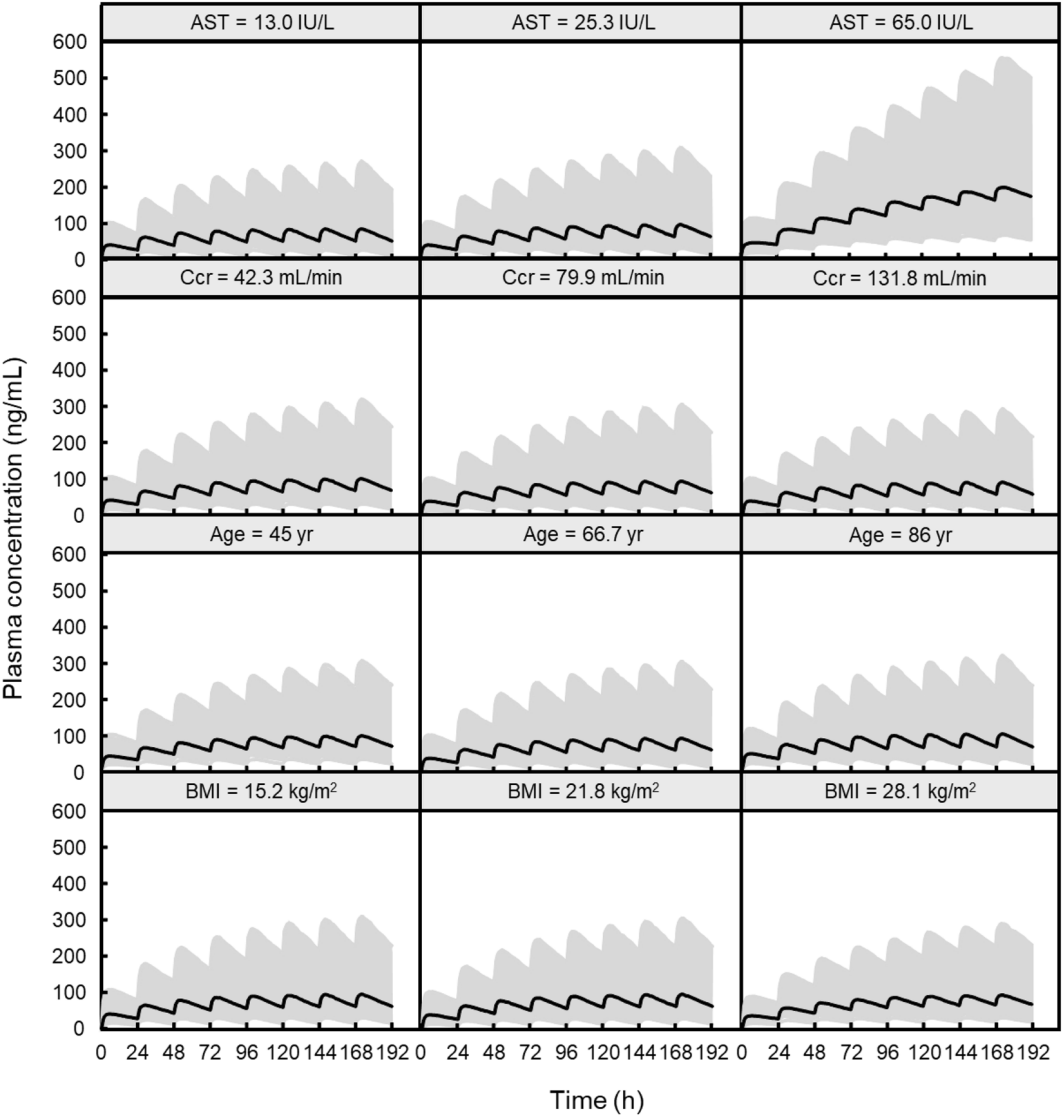
Simulated median afatinib plasma concentration-time profiles after oral administration of 40 mg/day afatinib for different scenarios regarding the median and 95% prediction interval of the 1000 simulated profiles for a typical reference patient (age 66.7 years; BMI 21.8 kg/m^2^; AST 25.3 IU/L; Ccr 79.9 mL/min). The solid lines show the median values, and the shaded area is the 95% prediction interval.

**Figure 5:**
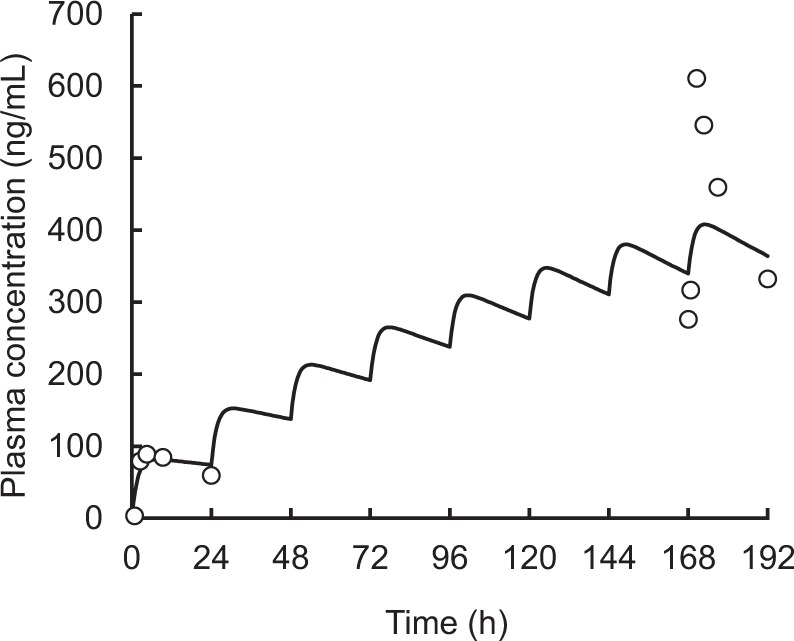
Simulated afatinib plasma concentration-time profiles after oral administration of 40 mg/day afatinib in patient #34 (age 63 years; BMI 22.3 kg/m^2^; AST 65.0 IU/L; Ccr 101.8 mL/min) using the final population PK model and *post hoc* estimates of pharmacokinetic parameters (ka 0.58 1/h; CL 3.1 L/h; V/F 465.7 L). The dots and solid lines represent the observed and simulated data, respectively.

Figure 3 shows the simulated population mean plasma concentration profiles of afatinib following administration at 40 mg/day for different AST values in a typical reference patient (age 66.7 years; BMI 21.8 kg/m^2^; Ccr 79.9 ml/min). High steady-state afatinib exposure was expected in patients with hepatic impairment. The 95% prediction interval profile for the typical patient indicated that hepatic impairment is a factor inducing large inter-individual variation in plasma concentration of afatinib. These simulated profiles also suggested that no other significant covariate (Ccr, Age, and BMI) notably affected the plasma concentration of afatinib following multiple administration (Fig. 4). The final model with *post hoc* estimates of PK parameters simulated the elevation of plasma concentration of afatinib following multiple doses in patient #34 with hepatic impairment (AST = 65 IU/L, ALT = 77 IU/L) (Fig. 5).

### Safety

Table S2 summarizes the toxicities associated with afatinib in all patients (see Supplementary Table S2). As expected, anorexia, diarrhea, skin complications, and stomatitis were the most frequently reported AEs. Grade 3 to 4 hematological toxicities (number; % of patients) included leukopenia (1; 2.9%) and neutropenia (1; 2.9%), whereas grade 3 to 4 nonhematological toxicities included anorexia (4; 11.8%), stomatitis (2; 5.9%), diarrhea (8; 23.5%), skin complications (3; 8.8%), pneumonitis (3; 8.8%), and infection (7; 20.6%).

Table 3 shows the relationship between the toxicities and trough plasma concentration of afatinib. Patients who experienced grade 3 anorexia, grade 3 stomatitis, grade 3 or 4 diarrhea, and grade 3 skin complications showed higher trough plasma concentrations of afatinib on day 8 than patients who experienced grade 0–2 AEs. However, a significant difference was found only in anorexia (p = 0.037).

**Table 3:** Relationship between the toxicities and dose modification, and trough plasma concentration of afatinib.

Characteristics	n	Trough plasma concentration (ng/mL)		
		Day 2	Day 8	
CTCAE grade				*p* Value
**Anorexia**				
0	24	20.28	52.03	
1	2	63.07	123.24	
2	4	42.37	49.98	
3	4	32.53	162.79	0.037
**Stomatitis**				
0	21	20.15	55.5	
1	5	32.41	93.98	
2	6	42.37	93.74	
3	2	36.33	180.87	0.117
**Diarrhea**				
0	5	48.14	50.24	
1	10	19.83	56.25	
2	11	28.05	58.4	
3	7	32.41	146.12	0.061
4	1	134.26	—	
**Skin complications**				
0	17	27.89	59.52	
1	9	24.3	58.8	
2	5	35.13	53.71	
3	3	29.92	141.78	0.117
**Dose modification**				
Dose reduction/interruption				
≦14days	8	26.51	115.6	
≦28days	13	27.89	103.32	0.027
All	22	31.17	79.74	
No change	10	20.15	45.46	
Dose escalation	1	4.89	8.6	

*p* value in Wilcoxon rank sum test.

*p* value for the comparison between grade 0–2 and grade 3–4 or reduction/interruption and no change/dose escalation.

A total of 22 patients required dose reduction or interruption of afatinib; 8 required it within 14 days, whereas 13 required it between days 15 and 28 (Table 3). The trough plasma concentration of afatinib in these 22 patients on day 8 were significantly higher than that in those who did not require dose reduction or interruption (p = 0.027).

## Discussion

We developed a population PK model based on plasma afatinib concentration data from Japanese NSCLC patients harboring EGFR mutation. Only one population PK model of afatinib has been reported so far and Freiwald *et al*. have reported population PK characteristics of afatinib in patients with breast cancer, head and neck squamous cell carcinoma or NSCLC using two-compartment model with first-order absorption and elimination^11^. In this study, a simple PK model, one-compartment model with first-order absorption and elimination, adequately described the PK profile of afatinib. A high inter-patient variability in PK parameters was observed, consistent with a previous report^11^. We estimated a lower population mean CL/F value (20.0 L/h) and higher *k*a value (0.60 1/h) relative to the results of the study by Freiwald *et al*. (42.3 L/h and 0.252 1/h, respectively). The effects of Ccr and AST on CL/F and the effect of BMI and age on V/F were significant covariates in Japanese NSCLC patients. Although Ccr was identified as the covariate of CL/F in the previous report^11^, our results also identified hepatic function marker as a covariate. Contrary to this finding, Freiwald *et al*. did not observe any significant effect of hepatic impairment on afatinib exposure; however, they determined that *a priori* dose adjustment based on sex, body weight, renal impairment, levels of performance score, alkaline phosphatase, lactate dehydrogenase, and total protein was not necessary. The different results are possibly due to the limited number of patients with hepatic impairment in their study (only 0.8% of all evaluable pharmacokinetic samples from patients with moderate impairment). Simulations using the final population PK model showed that hepatic impairment had a significant impact on the plasma concentrations of afatinib at steady state, but renal impairment, BMI, and age had no effect. This finding is consistent with a previous report describing that moderate-to-severe renal impairment has a minor effect on the PK of afatinib^12^. In contrast, Schnell *et al*. reported that mild to moderate hepatic impairment had no clinically relevant effect on the PK of a single dose of afatinib^13^. These results suggest that hepatic impairment may affect afatinib levels in plasma at steady state after repeated treatment, and AST levels measured during the pre-dosing period may be used to adjust the afatinib dose. Nonetheless, additional population analyses with a larger sample size of patients with hepatic impairment are required.

Sequist *et al*. reported a phase III study of afatinib or platinum-based doublet chemotherapy in NSCLC patients with EGFR mutation^5^. In their study, dose reduction to less than 40 mg/day was required for 52% of the participants, with 19% needing more than one dose reduction. Furthermore, high inter-patient variability was observed in the plasma concentrations of afatinib. Dose modification according to patient tolerability reduced excessive afatinib exposure. The inter-patient variability of pre-dose plasma concentration of afatinib in the 40 mg dose group decreased from 85.0% (day 1 of cycle two) to 66.5% (day 1 of cycle three). LUX-Lung 4 was a Japanese single-arm phase II trial of afatinib in patients previously treated with erlotinib and/or gefitinib. Patients received 50 mg/day afatinib orally in this study; 69.4% of patients required dose reduction to 40 mg/day and 35.5% of patients required further dose reduction to 30 mg/day^14^.

Furthermore, *post-hoc* analyses of LUX-Lung 3 and 6 reported the effect of afatinib dose reduction on AEs, PK, and PFS^15^. In these studies, afatinib trough plasma concentrations on day 22 were higher in patients whose dose was subsequently reduced to 30 mg than in those who remained on the 40 mg dose (geometric mean, 45.6 ng/ml vs 24.3 ng/ml). This result demonstrated that tolerability-guided dose adjustment allows to reduce treatment-related AEs without affecting treatment efficacy. Our study evaluated the PK of afatinib and investigated its relationship with AEs. The trough plasma concentration of afatinib on day 8 was significantly higher in the patients who required dose reduction or interruption than in those who did not (p = 0.0272). Dose reduction and discontinuation of afatinib appeared to be necessary in many cases, owing to toxicities. This dose discontinuation could lead to impairment of efficacy.

Yokoyama *et al*. conducted a phase II study of first line low dose afatinib in advanced NSCLC patients with EGFR mutations^16^. Afatinib was orally administered at the starting dose of 20 mg/day. If tolerated, this dose was increased in 10 mg increments up to 50 mg/day. The objective RR was 81.8% and 30.4% reported grade 3 or more AEs. The authors reported that low dose of afatinib at initial treatment with dose modification could be considered a better strategy than the treatment with standard dose. The dose–response relationship of cytotoxic anticancer drugs is important. An appropriate treatment efficacy cannot be expected from insufficient dose regimen.

The standard dose of EGFR TKIs is determined irrespective of body size. A retrospective study reported that the median PFS of patients with higher body surface area (BSA) was worse than that of those with lower BSA^17^. In contrast, Imai *et al*. reported that gefitinib efficacy in patients with EGFR-mutated advanced NSCLC did not differ according to BSA, body weight, or BMI. However, OS was higher in patients with both exon 19 deletion and low BSA than in patients without these characteristics^18^. These results suggested that BSA- and body weight-based dose adjustments could affect the treatment with EGFR TKIs. Unlike that of parenteral injection, the dose of oral tablets is difficult to adjust. However, the same dose for all patients seems to be related to differences not only in efficacy but also in toxicity. If the high plasma concentrations of afatinib are responsible for the toxicities, therapeutic drug monitoring (TDM) of plasma afatinib concentrations may help prevent toxicities associated to over-exposure.

There are some caveats in the present study. First, we did not investigate the potential of drug–drug interactions and genetic polymorphisms of drug metabolite. Antacid treatments decrease the serum concentrations of first-generation EGFR TKIs. Administration of acid-reducing agents and first-generation EGFR TKIs to healthy subjects decreases the area under the plasma drug concentration-time curve and the peak plasma concentration of first-generation EGFR TKIs^19^. The solubility of afatinib is high throughout the physiological pH range of 1–7.5, and therefore, no interactions with acid-reducing drugs are expected^20^. Gefitinib and erlotinib are metabolized primarily by cytochrome P450 (CYP)3A4, CYP3A5, and CYP1A1^21^. Therefore, concurrent use of these agents with CYP3A4 inhibitors, such as macrolide antibiotics, azoles, protease inhibitors, or grapefruit juice, may result in increased drug levels and toxicity. However, afatinib is not metabolized by CYP enzymes^22^. Afatinib is a substrate of P-glycoprotein and an inhibitor of P-glycoprotein and Breast Cancer Resistance Protein^22^. Therefore, concurrent therapy with P-glycoprotein inducers may decrease afatinib exposure. Recently, Hayashi *et al*. reported that genetic polymorphisms related to afatinib had influence on the pharmacokinetics in the early phase and were correlated with the severity of diarrhea^23^. Second, we did not investigate the effect of meals. According to the instruction in the package label, food should be avoided when administering afatinib^22^. In particular, a high-fat meal decreases afatinib exposure. In this study, afatinib was administered on an empty stomach, in accordance with the package label instructions. Third, few patients with severe hepatic and renal impairment, severe comorbidities, and poor overall condition, were a part of this study. The results of the current population PK analysis show hepatic function marker AST was identified as a covariate of CL/F, but ALT was not, which may be caused by limited sample size. Therefore, our results may not apply to these patients who have serious organ impairments. The fourth, there are several studies^13,24^ in which plasma afatinib concentrations were determined by using LS-MS or LC-MS/MS. In this study, we determined plasma afatinib concentrations with the HPLC system, since HPLC system is generally used at clinical sites due to ease of use and cost effectiveness. Though the sensitivity in determination is lower than the LS-MS or LC-MS/MS, plasma concentration of afatinib in patients is adequately measurable by HPLC system. Finally, our PPK model was not confirmed in a different cohort. Although we evaluated our final PPK model using a visual predictive check and nonparametric bootstrap analysis, a validation study is necessary to confirm the predictability of the final model in a validation cohort.

## Conclusions

The PK parameters of afatinib showed high inter-patient variability. The population PK analysis showed that hepatic impairment affected the PK parameters of afatinib. The trough plasma concentrations of afatinib on day 8 were higher in patients who required dose reduction or interruption due to dose-limiting toxicities than in those who did not. TDM is needed in cancer therapy with TKIs to reduce overexposure-related AEs.

## Patients and Methods

### Patients and treatment

The population PK analysis was performed using plasma afatinib samples obtained from 34 patients with NSCLC, who started afatinib therapy at the National Hospital Organization Kinki-Chuo Chest Medical Center and the Osaka Habikino Medical Center between August 2014 and May 2016. All patients met the following criteria: cytologically or histologically confirmed as EGFR mutation-positive NSCLC, with no history of treatment with afatinib in previous chemotherapy. Patients who had undergone gefitinib or erlotinib treatments in the past were included. Afatinib was orally administered at a dose of 40 mg/day once daily until disease progression or intolerable toxicity. Physicians were able to increase the afatinib dose up to 50 mg/day when they judged it was appropriate to increase the dose.

The protocol of this study was reviewed and approved by the institutional review board (IRB) at National Hospital Organization Kinki-Chuo Chest Medical Center, Osaka Habikino Medical Center, and Setsunan University. The methods were conducted in accordance with the ethical principles listed in the Declaration of Helsinki. Written informed consent was obtained from all participants prior to enrollment. This study was registered with the University Hospital Medical Information Network (UMIN) Clinical Trials Registry Identifier, UMIN000014181.

### Pharmacokinetic study

Blood samples were collected at 0.5–1, 2–3, 4–6, 8–12, and 24 h after oral administration of afatinib on day 1, and at 0, 0.5–1, 2–3, 4–6, 8–12, and 24 h after the dose on day 8. All blood samples were collected into potassium ethylenediaminetetraacetic acid-containing tubes and centrifuged immediately at 1800 × *g* (4 °C) for 10 min. The plasma samples obtained were stored at −80 °C until analysis. Plasma concentration of afatinib was determined using a high-performance liquid chromatography system (JASCO Corp., Tokyo, Japan). Further details of the assay have been described in our previous report^25^.

### Development of population pharmacokinetics model

Data was analyzed following the population approach and using a non-linear mixed-effect modeling software, namely Phoenix^®^ NLME^TM^ Version 7.0 (Certara USA, Inc., Princeton, NJ, USA). Non-compartment model analysis was initially performed to compare the PK parameters with those in previous studies^24,26^. The area under the plasma concentration–time curve from 0 h to 24 h after dosing (AUC_0–24h_) was calculated using the linear trapezoidal rule. The elimination half-life (t_1/2_) was determined by the terminal portion of the plasma concentration–time curve. To determine the population PK parameters and estimate their variability, the first-order conditional estimation with extended least squares (FOCE-ELS) method was used. Different PK models (1-, 2-, or 3-compartment model) were selected based on Akaike’s Information Criteria (AIC), goodness-of-fit plots, including observed (OBS) vs. individual predicted concentrations (IPRED), conditional weighted residuals (CWRES) vs. independent variable (IVAR), and CWRES vs. population predicted concentration (PRED), as well as coefficient of variation (CV) in parameter estimates. A drop of 2 in the AIC value was adapted as the cut-off criterion for PK model improvement. Different absorption models including lag time model were also selected because absorption of TKIs is usually complex. The models were compared based on these criteria, and a one-compartment model with first-order absorption was adapted as the final basic PK model.

Inter-individual variability was modelled exponentially. Different error models (including the additive, proportional, combined or power) were tested to describe the residual variability accounting for the discrepancies between observed and predicted concentrations. After developing the base population PK model, covariate candidates were evaluated for significance. The covariates were added to the population PK model using the stepwise program in Phoenix^®^ NLME^TM^ (i.e., forward addition (*p* < 0.01) and backward elimination (*p* < 0.001) methods) with −2Log-Likelihood values. The covariate candidates included in the current study were age, height, body weight, BMI, and levels of AST, ALT, serum creatinine (Cre), and Ccr. Individual Ccr values were determined using the Cockcroft-Gault equation. Various covariate effects (power or linear) were initially tested, and continuous covariates were modeled using a linear equation and centered around the mean value of the subjects.

### Model evaluation

The final population PK model was evaluated by a visual predictive check and nonparametric bootstrap analysis. For the visual predictive check, the 5^th^, 50^th^, and 95^th^ percentiles of plasma concentration of afatinib were simulated to obtain data sets (n = 1000) using the final model parameters. A nonparametric bootstrap procedure (n = 1000) was conducted to compare the parameters with the final model parameters estimated from the original data set, and to obtain the confidence intervals for the model parameters.

### Model-based simulation

The effects of obtained covariates on afatinib plasma concentration were evaluated using final population PK model-based simulations. The 5^th^, 50^th^, and 95^th^ simulated percentiles of afatinib plasma concentration, after oral administration of 40 mg afatinib once daily for 8 days, were simulated (n = 1000) using the obtained fixed- and random-effect parameters. The plasma concentration-time profile in individual patients was simulated using the final population PK model and *post hoc* estimates of individual PK parameters.

### Exposure-safety relationships

Toxicity was evaluated based on a numeric grading system, in accordance with the National Cancer Institute Common Terminology Criteria for Adverse Events (CTCAE), version 4.0. Two-group comparisons of trough plasma concentration on day 8 were performed using the Wilcoxon rank sum test. Patients were divided into two groups; a grade 0–2 AEs group vs a grade 3–4 AEs group, and a dose reduction/interruption group vs a no change/dose escalation group. The results were considered statistically significant for P < 0.05.

### Ethical approval

The current study was conducted in accordance with the ethical standards of the institutional research committee, and with the 1964 Helsinki declaration and its later amendments or comparable ethical standards.

## Supplementary information

Supplementary Information

## Data Availability

The datasets generated and analyzed during the current study are available from the corresponding authors on reasonable request.
