# Supplementary material for: Population pharmacokinetics of afatinib and exposure-safety relationships in Japanese patients with EGFR mutation-positive non-small cell lung cancer
Source: Sci Rep. 2019 Dec 3;9:18202. doi: 10.1038/s41598-019-54804-9 (PMC6890782; doi:10.1038/s41598-019-54804-9)
Supplement: Supplementary file 1 — Supplementary Information [file 41598_2019_54804_MOESM1_ESM.docx]

**Population pharmacokinetics of afatinib and exposure**-**safety relationships in Japanese patients with EGFR mutation-positive non-small cell lung cancer**

Keiko Nakao^1^, Shinji Kobuchi^2^, Shuhei Marutani^3^, Ayano Iwazaki^3^, Akihiro Tamiya^1^, Shunichi Isa^4^, Kyoichi Okishio^4^, Masaki Kanazu^5^, Motohiro Tamiya^6^, Tomonori Hirashima^7^, Kimie Imai^3^, Toshiyuki Sakaeda^2^, Shinji Atagi^4^*

^1^ Department of Internal Medicine, National Hospital Organization Kinki-Chuo Chest Medical Center, Osaka, Japan

^2^ Department of Pharmacokinetics, Kyoto Pharmaceutical University, Kyoto, Japan

^3^ Faculty of Pharmaceutical Sciences, Setsunan University, Osaka, Japan

^4^ Department of Thoracic Oncology, National Hospital Organization Kinki-Chuo Chest Medical Center, Osaka, Japan

^5^ Department of Thoracic Oncology, National Hospital Organization Osaka Toneyama Medical Center, Osaka, Japan

^6^ Department of Thoracic Oncology, Osaka International Cancer Institute, Osaka, Japan

^7^ Department of Thoracic Malignancy, Osaka Habikino Medical Center, Osaka, Japan,

***Corresponding author:**

Shinji Atagi

Department of Thoracic Oncology, National Hospital Organization Kinki-Chuo Chest Medical Center, Sakai, Osaka 591-8555, Japan

**Tel.:** +81-72-252-3021

**Fax:** +81-72-252-3041

**E-mail:** atagi.shinji.pn@mail.hosp.go.jp

Supplementary Table S1. Pharmacokinetic parameters of afatinib after oral administration at 40 mg/day in non-compartment analysis

| Pharmacokinetic parameters |  | Day 1 | | Day 8 | |
| --- | --- | --- | --- | --- | --- |
| *C_max_* (ng/mL) |  | 52.6 (64.2) | 106.4 (103.7) | |  |
| *T_max_* (h) |  | 4.1 (0.50–24.0) | 3.0 (0.50–22.0) | |  |
| *t_1/2_* (h) |  | 27.6 (65.4) | 38.3 (61.6) | |  |
| *AUC*_0-24h_ (ng∙h/mL) |  | 816.8 (68.4) | 1983.1 (103.1) | |  |
| *CL/F* (L/h) |  | 20.9 (111.4) | 6.6 (99.2) | |  |
| *Vd/F* (L) |  | 829.6 (70.6) | 363.0 (111.0) | |  |

Data are expressed as geometric mean [gCV(%)] or median (range)

*C_max_*, maximum drug concentration in plasma; *T_max_*, time to reach *C_max_*; *t_1/2_*, elimination half-life; *AUC*_0-24h_, area under the drug plasma concentration–time curve; *CL/F*, clearance of drug from plasma after oral administration; *Vd/F*, volume of distribution after oral administration; gCV(%), geometric coefficient of variation (%)

Supplementary Table S2. Toxicities associated with afatinib in patients (*n* = 34) with epidermal growth factor receptor (EGFR) mutation-positive non-small cell lung cancer

| Toxicities | Patients (n) | |
| --- | --- | --- |
|  | grade 1-2 | grade 3≦ |
| Leukopenia | 4 | 1 |
| Neutropenia | 5 | 1 |
| Anemia | 19 | 0 |
| Thrombocytopenia | 3 | 0 |
| Elevated AST | 14 | 0 |
| Elevated ALT | 15 | 0 |
| Elevated Cre | 8 | 0 |
| Anorexia | 6 | 4 |
| Nausea | 2 | 1 |
| Vomiting | 2 | 0 |
| Stomatitis | 11 | 2 |
| Fatigue | 3 | 0 |
| Diarrhea | 21 | 8 |
| Skin complications | 14 | 3 |
| Paronychia | 7 | 0 |
| Pneumonitis | 0 | 3 |
| Infection | 3 | 7 |
| Fever (non-infectious) | 1 | 0 |

*ALT,* alanine aminotransferase; *AST,* aspartate aminotransferase; *Cre,* serum creatinine.

**Supplementary Figure S1.** Relationship of all the candidate covariates.

Gender (male = 1, female = 2); Height (cm); Weight (kg); AST, aspartate aminotransferase (IU/L); ALT, alanine aminotransferase (IU/L); Cre, serum creatinine (mg/dL); Ccr, creatinine clearance (mL/min); BMI, body mass index (kg/m^2^)

**Supplementary Figure S2.** Plasma concentration-time profiles of afatinib in patients with EGFR mutation-positive non-small cell lung cancer.

0

100

200

300

400

500

600

700

0

24

168

192

Time (h)

Plasma concentration of afatinib (ng/mL)
